# A Two-to-Five Year Follow-Up of a Pediatric Acute-Onset Neuropsychiatric Syndrome Cohort

**DOI:** 10.1007/s10578-021-01135-4

**Published:** 2021-02-09

**Authors:** Caroline Gromark, Eva Hesselmark, Ida Gebel Djupedal, Maria Silverberg, AnnaCarin Horne, Robert A. Harris, Eva Serlachius, David Mataix-Cols

**Affiliations:** 1 Department of Clinical Neuroscience, Karolinska Institutet, Stockholm, Sweden; 2 Centre for Psychiatry Research, Stockholm Health Care Services, Region Stockholm, Stockholm, Sweden; 3 Department of Women’s and Children’s Health, Karolinska Institutet, Stockholm, Sweden; 4 Division of Pediatric Rheumatology, Karolinska University Hospital, Stockholm, Sweden; 5 Centre for Psychiatry Research, Child and Adolescent Psychiatry Research Center, Gävlegatan 22, 113 30 Stockholm, Sweden

**Keywords:** PANS, PANDAS, OCD, Tourette, Immunopsychiatry

## Abstract

Little is known about the long-term prognosis of children with pediatric acute-onset neuropsychiatric syndrome (PANS). Out of the 46 eligible patients from the Karolinska PANS cohort, 34 consented to participate in a follow-up (median 3.3 years). Participants underwent a thorough clinical evaluation and were classified according to their clinical course. Resulting groups were compared on clinical characteristics and laboratory test results. We observed significant reductions in clinician-rated PANS symptom severity and improved general function. Two patients were classified as remitted, 20 as relapsing–remitting, and 12 as having a chronic-static/progressive course. The latter group had an earlier onset, greater impairment and received more pharmacological and psychological treatments. Although remission was rare, the majority of children with PANS were significantly improved over the follow-up period but a non-negligible minority of patients displayed a chronic-static/progressive course and required additional treatments. The proposed definitions of flare and clinical course may be useful in future clinical trials.

**Supplementary Information:**

The online version of this article (10.1007/s10578-021-01135-4) contains supplementary material, which is available to authorized users.

## Introduction

Pediatric acute-onset neuropsychiatric syndrome (PANS) describes the acute onset or worsening of obsessive compulsive (OCD) symptoms and/or anorexia in temporal relation with other severe neuropsychiatric and somatic symptoms. Secondary signs include anxiety, emotional lability, irritability/aggression, regression, deterioration in school performance, sensory and motor abnormalities and somatic signs such as enuresis, sleep disturbance and pain [1]. The diagnosis of PANS requires an acute onset, but subsequent clinical courses may range from fully remitting after a single episode, to a waxing and waning course with intense exacerbations or flares, or even more chronic or progressive courses. Exacerbations or flares have been described in PANS since the initial definition of the syndrome, without being part of the diagnostic criteria [1]. In the acute phase, the symptoms are often severe and may lead to significant loss of function [2]. Psychiatric comorbidities are common and may need to be treated in parallel to the PANS-related symptoms [3, 4]. Pediatric autoimmune neuropsychiatric disorder associated with streptococcal infection (PANDAS) constitutes a sub-group of patients within the broader PANS construct, which requires the verified presence of a streptococcal infection temporally associated with the onset of symptoms [5].

While the psychiatric and somatic status of patients with PANS/PANDAS has been reasonably well described in cross-sectional studies, including high rates of pre-existing neuropsychiatric disorders and a strong association with autoimmune disease in both patients and first degree relatives, there is a shortage of long-term follow-up data of PANS/PANDAS cohorts [2, 3]. The available longitudinal data primarily originates from a handful of case reports and chart reviews, methods that not well suited for assessing the fraction of participants who may be remitting [6]. In a notable exception, Leon et al. described longitudinal outcomes for 33 patients with PANDAS who were followed up by telephone after participation in a randomized trial of intra-venous immunoglobulins (IVIG) compared to placebo, 0.5 to 4.8 years after trial completion [7]. At baseline, participants were required to meet the criteria for clinically significant obsessive–compulsive symptoms, in addition to at least three other comorbid neuropsychiatric symptoms. During the follow-up period, a majority (72%) of the patients had experienced at least one exacerbation of PANDAS symptoms, defined as a noticeable increase in a child’s previous PANDAS symptoms for a period of at least 24 h. A small fraction (12%) experienced clinically significant OCD-symptoms at follow-up, suggesting that the long-term prognosis of children with PANDAS was generally positive. However, a small proportion of cases (9%) had a more chronic course. A third of the children (33%) had received at least one new psychiatric diagnosis during the follow-up period, the most common being attention-deficit/hyperactivity disorder (ADHD) (18%). No face-to-face psychiatric or somatic evaluations or laboratory tests were conducted. To our knowledge, there are no published systematic follow-up data on patients with PANS, and thus their prognosis in the medium-to-long-term timeframe is currently unknown.

Given the paucity of available follow-up data on this patient group, we aimed to conduct a naturalistic follow-up of the patients included in a well-characterized Swedish cohort [3]. To minimize the risk of selectively following-up chronic patients in need of medical attention, we aimed to re-contact families regardless of whether they were still active in our clinic or not. Specifically, we conducted an assessment of current psychiatric and somatic health status, including laboratory tests, at the time of follow-up and investigated clinical characteristics that may influence disease course and prognosis in our cohort. An additional aim was to propose operational definitions of symptom flare and of various clinical courses of the syndrome that could be helpful in future research and clinical settings.

## Methods

### Clinical Setting

All study participants were recruited from a specialist OCD and related disorders outpatient clinic in Stockholm, Sweden, and had previously been included in the PANS cohort at Karolinska Institutet [3]. The clinic primarily receives referrals from Child and Adolescent Psychiatry Services (CAMHS) and pediatric services in the Stockholm region, but also from other parts of Sweden and the Nordic countries. The clinic has accepted PANS referrals since 2014.

All patients in the PANS cohort with a minimum of 2 years since inclusion were eligible for participation in the follow-up, regardless of whether they were still active patients in the clinic or not. Patients and parents/legal guardians gave written consent to participate in the initial cohort as well as the follow-up study, both approved by the Regional Ethics Review Board in Stockholm [reference number EPN 2015/1977-31/4 (2019-02132)].

### Clinical Evaluations

The evaluation at follow-up was a 2-h face-to-face assessment conducted by a child and adolescent psychiatrist. The assessment included a standardized patient- and parent interview focusing on current psychiatric and somatic health status, clinical change since the first visit, number of flares, potential triggers and course of disease, culture-verified and non-verified infections, previous and current medication, psychological treatments and family history of psychiatric and autoimmune or inflammatory disease. The information provided by the families was also verified against the patients’ electronic medical records. Questions about pre-school/school attendance referred to a time period of 3 months prior to the follow-up assessment. A medical examination for documentation of somatic signs was made, including height, weight, skin, heart, lungs, stomach, thyroid, lymph nodes, ears, throat, joints, neurology and motor function.

The following clinician-rated measures of symptoms and general function were employed:

The Children’s Global Assessment Scale (CGAS) is a widely used single-item measure of general functioning (ranging from 1 to 100), regardless of treatment and/or prognosis [8, 9]. The measurement should reflect the most impaired level of a specified time period of 1 month. Its psychometric properties have been extensively validated [10].

The Clinical Global Impressions-Severity scale (CGI-S) is a clinician-rated scale measuring the severity of a patient’s psychiatric illness on an 8 point single scale, ranging from *‘normal’* (score 1) to *‘extremely ill’* (score 7) [11]. The Clinical Global Impressions-Improvement scale (CGI-I), was used to assess the degree of clinical improvement at follow-up, relative to baseline, with scores ranging from *‘very much improved’* (score 1) to *‘very much worse’* (score 7) [11]. Both CGI-S and CGI-I have been validated and are widely used clinical outcome measures in psychiatry [12].

The Children’s Yale-Brown Obsessive Compulsive Scale (CY-BOCS) and Yale Global Tic Severity Scale (YGTSS) are the gold standard instruments to quantify the severity of OCD and tic disorder symptoms, respectively [13–15]. They are routinely employed in both clinical practice and clinical trials and have excellent psychometric properties [14, 16, 17].

Baseline data on age at symptom onset, comorbidity at onset, Children’s Global Assessment Scale (CGAS) and Clinical Global Impressions-Severity scale (CGI-S) scores were extracted from the baseline clinical assessment, published in the original description of the cohort [3].

### Operational Definitions of Flare and Clinical Course

Based on our clinical experience with the patient group, we developed the following a priori operational definition of symptom flare:

We defined *flare* as worsening of PANS-related symptoms and/or loss of function (CGI-S equal to or > 4) for longer than 4 days, irrespective of treatment given. The time period of 4 days was chosen because, according to our clinical experience, medical treatments given to treat flares frequently give effect by this time.

Our definitions of *clinical course* were as follows:

1. Remitting course: Patients who experienced no PANS symptoms for the last 12 months.
2. Relapsing–remitting course: Patients who experienced at least one flare during the last 12 months, but who had been in remission > 50% of the time for the last 12 months.
3. Chronic-static/progressive course: Patients fulfilling criteria for flare > 50% of the time for the last 12 months.

### Laboratory Analyses

The laboratory protocol was the same one developed by the team for regular clinical use [3]. It includes basic blood measurements of C-reactive protein (CRP), Erythrocyte Sedimentation Rate (ESR), hemoglobin, complete blood count (CBC), thyroid tests, indicators for liver and kidney disease, vitamin D levels, ferritin, celiac test and inflammatory and rheumatological markers such as protein fractions, immunoglobulin G, A and M, IgG subclasses, antinuclear antibodies (ANAs), serum amyloid A (SAA) and interleukin (IL)-1-β, IL-6, IL-8, IL-10 and tumor necrosis factor (TNF)-α. A throat culture was taken at the same time. The reference values used were those used clinically by the Karolinska laboratory [18].

### Statistical Analysis

Statistical analysis was conducted using STATA software (version STATA/IC15.1 for Mac, StataCorp LLC, Texas, USA). Analyses were largely descriptive in nature. When comparing the various clinical course sub-groups of patients within the cohort, t-tests were employed for continuous variables with parametric distributions and Wilcoxon rank-sum tests or Wilcoxon signed-rank tests for non-parametric distributions. Chi-square tests were employed for proportions. A p-value below 0.05 was considered statistically significant.

## Results

### Demographic and Clinical Characteristics

**Fig. 1:**
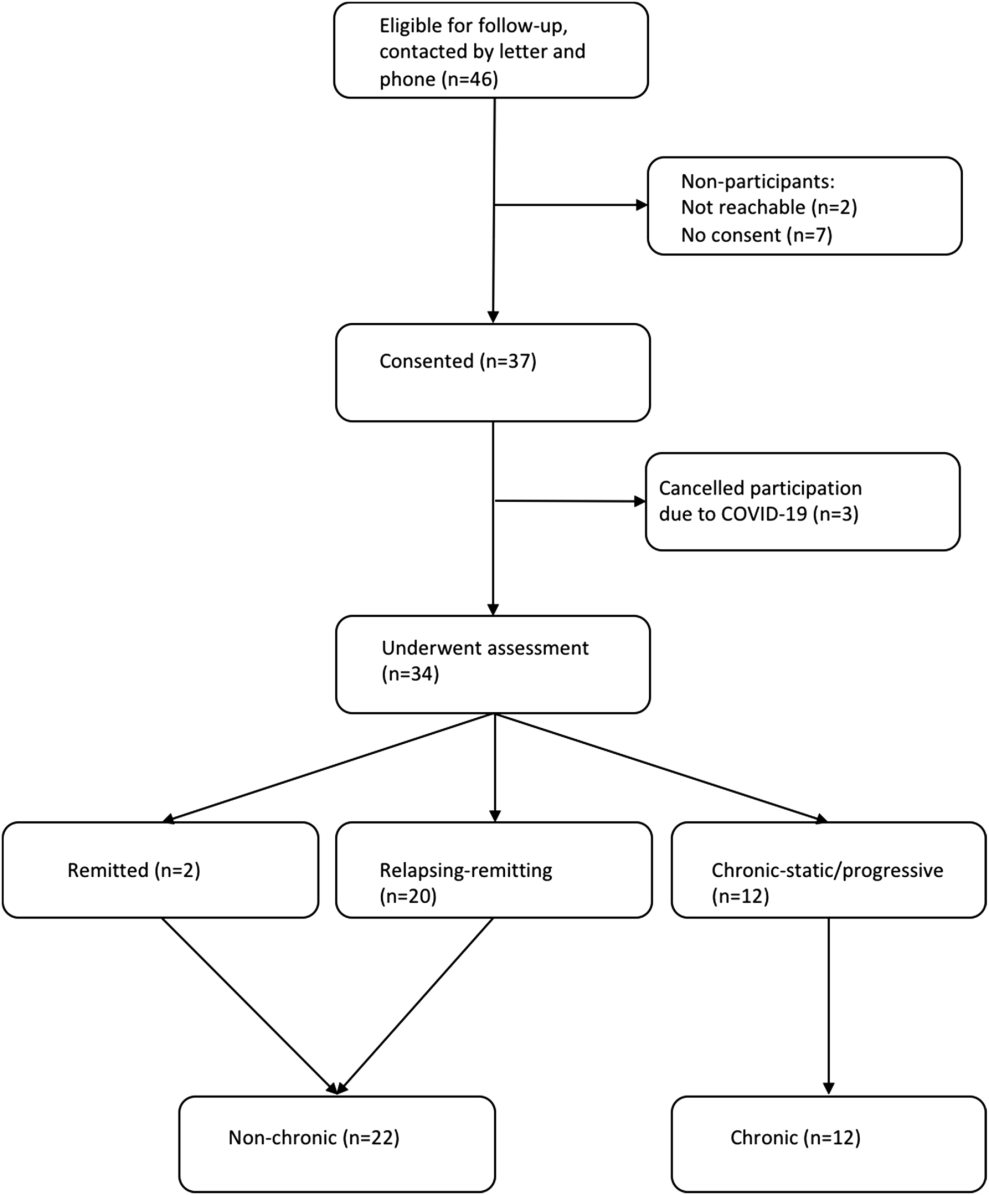
Flow chart of participant inclusion, and groupings based on clinical course

Out of the 46 patients eligible for follow-up, 34 consented to participate (Fig. 1)*.* The main reasons for attrition were that the families no longer wished to participate in research or that they canceled their participation due to the ongoing COVID-19 pandemic. There were no statistically significant differences in baseline characteristics between those who were available or unavailable at follow-up (Table S1). Median age at disease onset was 6.6 years (range 3–11.5), median age at follow-up was 11.5 years (range 6.7–17.1) and 19 (56%) of the participants were male. Median time since symptom onset was 4.8 years (range 3–9.2) and the median follow-up time was 3.3 years (range 2.3–4.9).

The number of flares over the last 12 months prior to the follow-up assessment ranged from 0 to 3 (median = 1). Twenty participants (59%) reported no verified infections (positive culture or positive clinical assessment) during the 12 months prior to follow-up, ten (29%) reported one, three (9%) reported two and only one (3%) reported four. The number of reported unverified infections (parent-reported) during the same period was much higher, ranging from 0 to 8 (median = 2.5).

Only two participants (6%) had a previous neuropsychiatric diagnosis (ASD) at baseline. During the follow-up time, another 13 (38%) received a neuropsychiatric diagnosis (nine (26%) ADHD, three (9%) ASD and one (3%) intellectual disability). Eleven participants (32%) had a comorbid condition commonly considered to have an autoimmune or inflammatory etiology, of which four (12%) were newly diagnosed during the follow-up period. These conditions included severe asthma, severe atopic eczema, severe and multiple nutritional allergies, celiac disease, autoimmune thyroiditis, postinfectious arthritis, and Henoch-Schönlein’s purpura. Twenty-eight participants (82%) had a family history of inflammatory or autoimmune disease and 27 (79%) had a family history of psychiatric disorder. During the 3 months prior to the follow-up interview, six participants (18%) had missed more than one school day per week on average. For a full summary of the sample’s socio-demographic data, see Table S2.

For the 34 patients included in the follow-up, the median CGAS score had increased from 53 (range 54–70) at baseline to 61 (range 28–80) at follow-up, a statistically significant difference (z = − 3.59, p < 0.001). The wide range of scores is suggestive of substantial heterogeneity within the cohort.

**Fig. 2:**
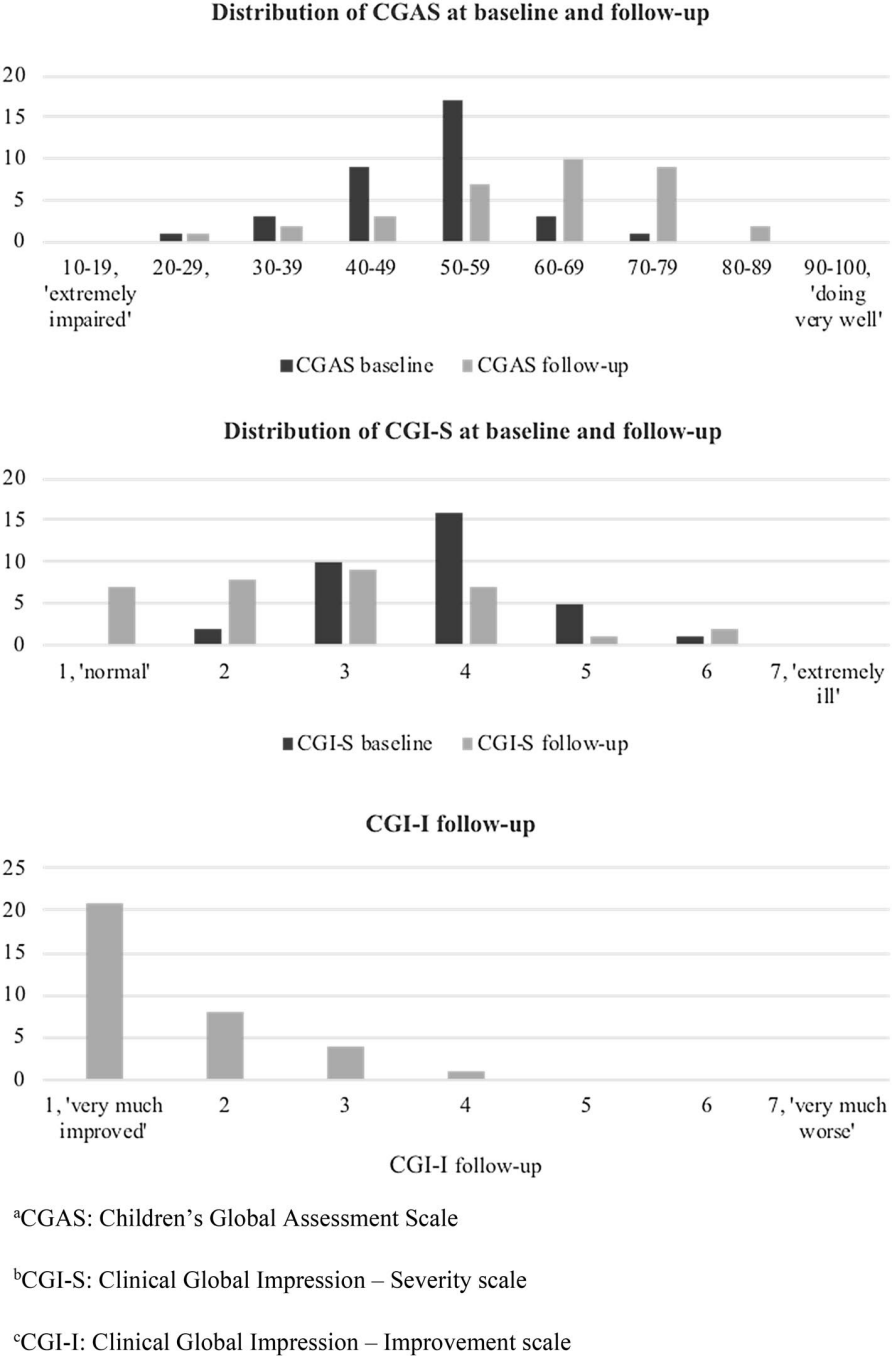
Distribution of clinician-rated CGAS^a^ and CGI-S^b^ scores at baseline and follow-up, and of CGI-I^c^ scores at follow-up. ^a^CGAS: Children’s Global Assessment Scale. ^b^CGI-S: Clinical Global Impression—Severity scale. ^c^CGI-I: Clinical Global Impression—Improvement scale

Similarly, the CGI-S scores had decreased from a median moderate level of severity 4 (range 2–6) to a mild level 3 (range 1–6), a statistically significant difference (z = 3.70, p < 0.001). The median CGI-I score was 1 (range 1–4), and 29 participants (85%) were rated as being ‘much improved’ or ‘very much improved’, indicating substantial improvements relative to baseline at the group level (Fig. 2).

### Psychiatric Symptoms and Somatic Signs at Follow-Up

The most common reported symptoms at follow-up were obsessive–compulsive symptoms (62%) and tics (50%). About a third of the patients reported anxiety (35%), hyperactivity/impulsivity (35%), behavioral difficulties (32%), depressive symptoms (29%), sleep disorder (29%) and tiredness/fatigue (29%).

The median CY-BOCS score at follow-up was 8 (range 0–30). Only five participants (15%) had a CY-BOCS score above 15, corresponding to the minimum entry severity criterion in clinical trials of OCD. Though half of the participants reported tics at follow-up, the YGTSS scores were low (median 4.5, range 0–65). Only two (6%) scored above 30, indicating that clinically relevant tic disorder was uncommon at follow-up. Approximately a third of the participants (36%) had some kind of abnormality in the somatic assessment (skin abrasions and eczema being the most common ones), but only a minority of these findings was clinically meaningful or actionable (Table 1).

**Table 1:** Psychiatric symptoms and somatic signs at follow-up (n = 34)

	n	%
Obsessive–compulsive symptoms	21	62
CY-BOCS score > 15	5	15
Tics	17	50
YGTSS scores > 30	2	6
Hyperactivity/impulsivity	12	35
Anxiety symptoms	12	35
Behavioral difficulties	11	32
Depressive symptoms	10	29
Sleeping difficulties	10	29
Tiredness/fatigue	10	29
Pain	8	24
Cognitive difficulties	6	18
Eating disorder symptoms	5	15
Urinary problems	2	6
Perceived changes in personality	1	3
Abnormalities in somatic assessment	12 (n total = 33)	36
Skin abrasions, eczema, psoriasis	9 (n total = 33)	27
Choreatic movements	2 (n total = 33)	6
Otitis	2 (n total = 33)	6
Tonsillitis	1 (n total 33)	3

*CY-BOCS* Children’s Yale-Brown Obsessive Compulsive Scale, *YGTSS* Yale Global Tic Severity Scale

### Treatments Received During the Follow-Up

Eighty-five percent of the participants had received some kind of medication over the last 12 months prior to the follow-up assessment. Melatonin was the most common prescription (47%), followed by non-steroidal anti-inflammatory drugs (NSAIDs) (44%), antibiotics (41%), guanfacine (24%) and selective serotonin re-uptake inhibitors (SSRIs) (21%). Only one participant (3%) had been prescribed antipsychotics. None of the antibiotics prescribed from our clinic were long-term or prophylactic prescriptions, but one participant was on long-term antibiotic prophylaxis, which had been initiated at another clinic. The rest of the antibiotic prescriptions were all short-term courses of treatment prescribed in association with a verified or highly suspected infection. Immunomodulatory drugs such as cortisone and IVIG were less common (12% each).

A majority (59%) of the participants had received some kind of psychological treatment or parental intervention in the 12 months prior to the follow-up assessment. Thirty-eight percent had received CBT and 44% other interventions, such as parental strategies, parental groups and counseling (Table 2).

**Table 2:** Pharmacological and psychological interventions received during the 12 months prior to the follow-up appointment (n = 34)

	n	%
Any medication	29	85
Melatonin	16	47
NSAIDs	15	44
Antibiotics	14	41
Guanfacine	8	24
SSRI	7	21
Cortisone	4	12
IVIG	4	12
Stimulants	3	9
Neuroleptics	1	3
Any psychological treatments/interventions	20	59
Other interventions (parental strategies, groups, counselling)	15	44
CBT	13	38

*NSAIDs* non-steroidal anti-inflammatory drugs, *SSRI* selective serotonine re-uptake inhibitor, *IVIG* intra-venous immunoglobulins, *CBT* cognitive behavioral therapy

### Laboratory Findings

Twenty-seven of the 34 participants gave samples for laboratory tests taken at the time of follow-up. The missing laboratory tests were due to the COVID-19 restrictions in Sweden at the time of the study, preventing some of the participants to access the Karolinska laboratory facilities. Complete blood count (CBC) abnormalities and protein fractions abnormalities were common (78% and 74%, respectively). Six participants (22%) had complement abnormalities. Almost a fifth (19%) had elevated thyroid peroxidase antibodies (anti-TPO), 15% had thyroid stimulating hormone (TSH) abnormalities and 11% had low thyroxine (T4). Two participants (7%) had positive ANAs. Only one (4%) had low IgG, but 41% had IgG sub-class deficiencies. Thirty-six per-cent had elevated IL-1-β, 20% elevated IL-10 and 19% elevated TNF-α. None of the other measured cytokines were elevated. Twelve percent of the sample had a positive streptococci throat culture (Table 3). With the exception of the positive streptococci cultures, which required management with antibiotics, the remainder of the laboratory findings did not require specific medical treatments. Thyroid abnormalities initiated further laboratory screening, but were not sufficiently severe to require immediate medical intervention.

**Table 3:** Laboratory tests taken at follow-up (n = 27)

	n	%
Protein fractions abnormalities (incl CRP)	20	74
CBC abnormalities	18 (n total = 26)	69
IgG sub-class deficiencies	11	41
Elevated IL-1-β	9 (n total = 25)	36
Complement abnormalities	6	22
Low vitamin D	6	22
Low IgA	6	22
Elevated IL-10	5 (n total = 25)	20
Elevated TNF-α	5 (n total = 26)	19
Elevated anti-TPO	5	19
TSH abnormalities	4	15
Positive throat culture	3 (n total = 25)	12
Low T4	3	11
Elevated IgM	3	11
Positive ANA	2	7
Elevated ESR	1 (n total = 26)	4
Elevated CRP	1	4
Elevated SAA	1	4
Low IgG	1	4
Elevated transglutaminase antibodies	0	0
Elevated IL-6	0 (n total = 26)	0
Elevated IL-8	0 (n total = 26)	0

*CBC* complete blood count, *IgG* immunoglobulin G, *IL-1-β* interleukin 1 β, *IgA* immunoglobulin A, *IL-10* interleukin 10, *TNF-α* tumor necrosis factor α, *Anti-TPO* thyroid peroxidase antibodies, *TSH* thyroid stimulating hormone, *T4* thyroxine, *IgM* immunoglobulin M, *ANA* antinuclear antibodies, *ESR* erythrocyte sedimentation rate, *CRP* C-reactive protein, *SAA* serum amyloid A, *IL-6* interleukin 6, *IL-8* interleukin 8

### Groupings Based on Clinical Course

Using the a priori operational definitions of flare and disease course, and based on all available information at the time of follow-up assessment, two participants (6%) were classified as having remitted, 20 (59%) as relapsing–remitting, and 12 (35%) as having a chronic-static/progressive course. Due to the insufficient number of participants in full remission, we recoded the groups into a non-chronic group (remitting + relapsing–remitting groups) and a chronic group (chronic-static/progressive group). Twenty-two participants (65%) were thus classified as being non-chronic and 12 (35%) as being chronic.

There was no gender difference between the non-chronic and chronic groups (χ^2^ = 0.5, p = 0.62). Interestingly, age at baseline and follow-up were both significantly lower in the chronic compared to the non-chronic group (z = 2.36, p = 0.02 and z = 2.04, p = 0.04, respectively), while the follow-up time was similar between the two groups (z = − 0.25, p = 0.8). There was a tendency towards a higher autoimmune or inflammatory comorbidity in the chronic group (50%) compared to the non-chronic group (23%), but this difference was not significant (χ^2^ = 2.64, p = 0.1). The same was true for having received a neuropsychiatric diagnosis during the follow-up time (seven participants (58%) compared to six (27%), χ^2^ = 3.17, p = 0.08). The median number of flares in the year prior to the follow-up interview was 0.5 (range 0–3) in the non-chronic group and 1 (range 0–3) in the chronic group, but the difference was not statistically significant (z = 1.22, p = 0.22). There were no between-group differences regarding family history of psychiatric or autoimmune and inflammatory disease (χ^2^ = 0.62, p = 0.64 and χ^2^ = 0.01, p = 0.91, respectively) (Table S2).

At baseline, the non-chronic and chronic groups had similar symptom severity (CGI-S scores), but the chronic course group had a significantly higher impairment (lower CGAS scores) (z = 2.08, p = 0.04). At follow-up, participants in the chronic course group remained significantly more impaired on the CGAS (z = 3.54, p < 0.001) and had significantly higher CGI-S scores (z = − 3.88, p < 0.001). As expected, the chronic course group had improved significantly less on the CGI-I than the non-chronic group (z = − 2.08, p = 0.04). School attendance was also lower in the chronic course group, with five out of 12 (42%) missing more than one school day per week on average, compared to only one out of 22 (5%) in the non-chronic course group (χ^2^ = 7.36, p = 0.01) (Table S2).

At follow-up, the two groups did not significantly differ in the severity of obsessive–compulsive or tic symptoms (CY-BOCS and YGTSS scores, respectively). However, the chronic course group had higher rates of hyperactivity/impulsivity (χ^2^ = 12,8, p < 0.001), anxiety (χ^2^ = 7.99, p = 0.01), behavioral difficulties (χ^2^ = 5.72, p = 0.02), depressive symptoms (χ^2^ = 7.47, p = 0.01), sleep disorder (χ^2^ = 12.4, p < 0.001), eating disorder (χ^2^ = 5.13, p = 0.02) and urinary problems (χ^2^ = 3.9, p = 0.05) than did the non-chronic course group. Abnormalities in the somatic assessment were more frequent in the chronic course group, but this difference was not statistically significant (χ^2^ = 1.52, p = 0.22) (Table S3).

All of the participants in the chronic course group had received medical treatment during the last 12 months. In this group, nine participants (75%) had received NSAIDs, nine (75%) melatonin, eight (67%) antibiotics and seven (58%) guanfacine. Four participants (33%) had received IVIG and three (25%) cortisone. About two thirds (77%) of participants in the non-chronic course group had received medication during the preceding 12 months, but this difference was not statistically significant. Compared to the non-chronic group, the prescription rates in the chronic group were significantly higher for IVIG (χ^2^ = 8.31, p < 0.001), NSAIDs (χ^2^ = 7.17, p = 0.01), antibiotics (χ^2^ = 4.97, p = 0.03), guanfacine (χ^2^ = 12.49, p < 0.001) and melatonin (χ^2^ = 5.81, p = 0.02). Similarly to medical treatments, the frequency of psychological/parental interventions was significantly higher in the chronic compared to the non-chronic course group (χ^2^ = 4.60, p = 0.03) (Table S4).

Levels of IL-1-β and TNF-α were both elevated in the chronic compared to the non-chronic course group (χ^2^ = 7.77, p = 0.01 and χ^2^ = 11.69, p < 0.001, respectively). IgM was also elevated in the chronic course group (χ^2^ = 6.75, p = 0.01). There were no other significant between-group differences detected in any of the laboratory analyses (Table S5).

### Post-hoc Analyses Comparing Laboratory Tests in Asymptomatic and Symptomatic Patients

Because of the relapsing–remitting nature of PANS, it is pertinent to further consider the symptomatic status of the patient when interpreting the results of the laboratory tests. In a post-hoc analysis, the participants were divided into an asymptomatic and a symptomatic group, depending on whether or not they presented active PANS symptoms at the time of follow-up.

Twenty participants (59%) presented with active PANS symptoms at the time of follow-up. Similar to the comparison between chronic and non-chronic participants, levels of IL-1-β and TNF-α were also significantly elevated in the symptomatic compared to asymptomatic participants (χ^2^ = 4.89, p = 0.03 and χ^2^ = 3.87, p = 0.05, respectively). IgM was slightly elevated in symptomatic participants, but the difference was not statistically significant (χ^2^ = 2.32, p = 0.13). None of the asymptomatic participants had a positive throat culture (Table S5).

## Discussion

We prospectively followed 34 patients for a period of 2-to-5 years, regardless of whether they were still active patients at our clinic or not. The latter was particularly important to limit the risk of selectively following-up chronic patients in need of medical attention. The main finding was that, although full remission was rare, the majority of children with PANS were significantly improved at the time of follow-up. Approximately 85% of the participants had much or very much improved PANS symptoms. Only a small proportion (15%) had clinically significant obsessive–compulsive symptoms at follow-up. Accordingly, the use of SSRIs and antipsychotics was relatively rare at follow-up. However, more than a third of the patients were classed as having a chronic course, and were still experiencing disabling symptoms requiring additional pharmacological and psychological interventions.

Clinician-rated global functioning, disease severity and improvement scales all indicated significant improvement during the follow-up time, though patients and parents frequently reported persisting symptoms influencing everyday life. This apparent discrepancy between clinician and subjective ratings may suggest that our instruments do not capture the full extent of the patients’ difficulties. Alternatively, clinicians and families may interpret PANS symptoms differently. This is an important topic that requires further investigation, as it is critical for the design of clinical trials and the choice of appropriate outcome measures.

A striking finding was that 38% of the participants had received an additional neuropsychiatric diagnosis during the follow-up period. Even though our naturalistic sample includes PANS patients with co-occurring autism and other severe psychiatric symptoms, thereby comprising a more complex patient group, the findings are similar to those of the Leon et al. PANDAS cohort [7]. They also reported that 33% of the participants had received a psychiatric diagnosis at follow-up, ADHD being the most common one. This highlights the importance of regular neuropsychiatric assessments in this patient group. The identification and correct diagnosis of these neuropsychiatric syndromes will facilitate the deployment of evidence-based interventions for specific problems, alongside any additional interventions required for PANS. An exclusive focus on PANS symptoms risks missing opportunities for intervention in other areas.

In our clinic, all suspected PANS patients are routinely evaluated with extensive somatic assessments and laboratory analyses, which are both time-consuming and costly. At intake, these procedures may be important in order to exclude other, potentially treatable, conditions such as infections, autoimmune thyroiditis, celiac disease, psoriasis or systemic lupus erythematosus (SLE). We previously reported baseline data for the cohort, showing high levels of somatic signs at intake (61% exhibiting skin abnormalities such as eczema or abrasions; 46% exhibiting signs of ear, nose or throat infections; and 23% showing neurological abnormalities including choreatic movements) [3]. However, at follow-up, the somatic findings were much rarer and often did not require a specific treatment. This suggests that the value of such a comprehensive assessment at follow-up may be limited, at least in routine clinical care. We suggest to mainly focus on signs of current infection. Because of the similarities between PANS and Sydenham’s chorea/rheumatic fever, we believe it is also advisable to routinely perform a heart auscultation, in order to rule out any murmurs indicative of heart inflammation.

Published data of inflammatory markers in PANS patients are scarce and difficult to interpret, since the level of disease activity is rarely rated at the time of testing. Our findings indicate that thyroid abnormalities may be more frequent at follow-up (anti-TPO 19%, TSH abnormalities 15% and low T4 11%) than at baseline (anti-TPO 11%, TSH abnormalities 10% and low T4 0%) [3]. IL-1-β and TNF-α levels were significantly elevated in both symptomatic patients and chronic-static/progressive patients. Interestingly, none of the patients in the initial cohort had elevated IL-1-β and/or TNF-α levels at baseline [3]. In a naturalistic study describing the characteristics of the first 47 consecutive patients at the Stanford PANS clinic, Frankovich et al. reported positive Anti-Nuclear Antibodies (ANAs) in 28% of the patients fulfilling PANS criteria [2]. At baseline, we recorded positive ANAs in 17% of the patients [3], and at follow-up only in 7%. In the absence of robust biomarkers for PANS, these findings clearly indicate that the value of a of full laboratory work-up for clinical practice is currently limited. We suggest the full analyses should include measurement of CBC, thyroid, liver and kidney markers, inflammatory markers, IgG (including sub-classes), IgA and IgM, ANA, celiac test and throat culture at disease onset or when evaluating the need of potential immunomodulatory treatments. A smaller number of clinically relevant tests will suffice during the follow-up. These tests should be chosen based on current signs of infection, abnormalities in the initial work-up, comorbidities or ongoing pharmacological treatments.

A novel aspect of our work is that we propose operational definitions of flare and clinical course in PANS, based on our long experience with this patient group. Previous definitions of flare and clinical course in patients with PANS/PANDAS are difficult to use in practice. Leon et al.defined a flare as a noticeable increase in a child’s previous PANDAS symptoms for a period of at least 24 h [7], which we consider problematic because this may risk capturing the natural waxing and waning of symptoms rather than a true flare. In a chart review of 218 consecutive patients at the Stanford PANS Clinic, Brown et al. defined a flare as an acute neuropsychiatric deterioration meeting strict PANS or PANDAS criteria, without the requirement of a specified time period [19], We believe that our proposed definition of a flare is more precise and, in our experience, easier to use in the clinic.

Regarding previous definitions of clinical course, Brown et al. employed the following definitions: relapsing–remitting with flares and an approximate return to baseline without need for immunomodulatory treatment; chronic-static with unchanging symptoms lasting at least 9 months; and progressive as a chronic course worsening in intensity over time [19]. These definitions may be ambiguous, as a patient with a flare resolved after administration of an immunomodulatory treatment will be difficult to classify as either relapsing–remitting or chronic. Our proposed definitions will hopefully enable classification of different disease courses regardless of treatment given, an essential factor in the research setting and clinical practice alike.

Despite the small sample size, our proposed definitions of flare and clinical course seemed to meaningfully distinguish the chronic and non-chronic course groups at follow-up regarding their level of impairment and subsequent need of healthcare resources. The groups also differed in some of the laboratory analyses (IL-1-β, TNF-α and IgM), but their clinical significance is currently unclear. However, as this represents a proinflammatory immune response, and there was also a link to autoimmunity (also caused by proinflammatory immune responses), further investigation of relative immune activation sensitivity is warranted to further explore patient subgroups. The chronic course group had an earlier onset, but follow-up time was comparable across the two groups; thus the natural course of the disorder is unlikely to explain the observed differences. Interestingly, the two groups had similar symptom severity at baseline but the chronic course group had significantly lower CGAS scores (i.e. more impairment), suggesting that both early onset and impaired function may potentially be useful predictors of chronicity in this patient group. The small sample size prevents further analyses of clinical characteristics predictive of disease course and prognosis, but we observed a trend towards higher rates of autoimmune or inflammatory comorbidity in the chronic course group. The identification of such predictive and prognostic factors should be a priority of future research with larger samples.

To our knowledge, this is the first naturalistic long-term follow-up study of a well-characterized PANS cohort. The main limitation of the study is the small sample size. The follow-up assessments coincided with the COVID-19 pandemic, resulting in an even smaller inclusion rate when previously consented participants wished to avoid visiting the clinic solely for research purposes. We had a particularly high data loss on the laboratory tests due to limited possibilities to travel to the laboratory to provide a blood sample. The baseline characteristics of the participants who were not available at follow-up were comparable to those of the participants who were available, somewhat mitigating this limitation. Finally, some of the measures administered at follow-up (e.g. CYBOCS and YGTSS) were not available at baseline, precluding certain longitudinal analyses.

## Summary

This long-term follow-up study showed that, although full remission was rare, the majority of children with PANS were significantly improved over a mean 3-year follow-up period. However, a non-negligible minority of patients displayed a chronic-static/progressive course and required additional treatments. Interestingly, these treatments seldom concerned OCD or tics, as these symptoms seemed to remit during the follow-up in a large proportion of participants. The proposed operational definitions of flare and clinical course appeared to meaningfully distinguish the chronic and non-chronic groups at follow-up regarding their level of impairment and subsequent need of healthcare resources and may be useful in clinical practice, in future clinical trials, and in the development of treatment guidelines.

## Supplementary Information

Below is the link to the electronic supplementary material.

Electronic supplementary material 1 (DOCX 13 kb)

Electronic supplementary material 2 (DOCX 16 kb)

Electronic supplementary material 3 (DOCX 14 kb)

Electronic supplementary material 4 (DOCX 14 kb)

Electronic supplementary material 5 (DOCX 16 kb)

## Data Availability

The data are pseudonymised according to national (Swedish) and EU legislation, and cannot be anonymised and published in an open repository. The data can be made available upon reasonable request on a case by case basis according to the current legislation and ethical permits.

## References

[1] Swedo S, Leckman J, Rose N (2012). From research subgroup to clinical syndrome: modifying the PANDAS criteria to describe PANS (pediatric acute-onset neuropsychiatric syndrome). Pediatr Therapeut.

[2] Frankovich J, Thienemann M, Pearlstein J, Crable A, Brown K, Chang K (2015). Multidisciplinary clinic dedicated to treating youth with pediatric acute-onset neuropsychiatric syndrome: presenting characteristics of the first 47 consecutive patients. J Child Adolesc Psychopharmacol.

[3] Gromark C, Harris RA, Wickström R, Horne A, Silverberg-Mörse M, Serlachius E (2019). Establishing a pediatric acute-onset neuropsychiatric syndrome clinic: baseline clinical features of the pediatric acute-onset neuropsychiatric syndrome cohort at Karolinska Institutet. J Child Adolesc Psychopharmacol.

[4] Thienemann M, Murphy T, Leckman J, Shaw R, Williams K, Kapphahn C (2017). Clinical management of pediatric acute-onset neuropsychiatric syndrome: part I-psychiatric and behavioral interventions. J Child Adolesc Psychopharmacol.

[5] Swedo SE, Leonard HL, Garvey M, Mittleman B, Allen AJ, Perlmutter S (1998). Pediatric autoimmune neuropsychiatric disorders associated with streptococcal infections: clinical description of the first 50 cases. Am J Psychiatry.

[6] Sigra S, Hesselmark E, Bejerot S (2018). Treatment of PANDAS and PANS: a systematic review. Neurosci Biobehav Rev.

[7] Leon J, Hommer R, Grant P, Farmer C, D'Souza P, Kessler R (2018). Longitudinal outcomes of children with pediatric autoimmune neuropsychiatric disorder associated with streptococcal infections (PANDAS). Eur Child Adolesc Psychiatry.

[8] Endicott J, Spitzer RL, Fleiss JL, Cohen J (1976). The global assessment scale. A procedure for measuring overall severity of psychiatric disturbance. Arch Gen Psychiatry.

[9] Shaffer D, Gould MS, Brasic J, Ambrosini P, Fisher P, Bird H (1983). A children's global assessment scale (CGAS). Arch Gen Psychiatry.

[10] Schorre BE, Vandvik IH (2004). Global assessment of psychosocial functioning in child and adolescent psychiatry. A review of three unidimensional scales (CGAS, GAF, GAPD). Eur Child Adolesc Psychiatry.

[11] Guy W (1976) ECDEU Assessment manual for psychopharmacology—revised. U.S. Department of Healt EaW, Public Health Service, Mental Health Administration. In: Rockville MD (ed) NIMH Psychopharmacological Research Branch, Division of Extramural Research Programs

[12] Berk M, Ng F, Dodd S, Callaly T, Campbell S, Bernardo M (2008). The validity of the CGI severity and improvement scales as measures of clinical effectiveness suitable for routine clinical use. Journal of evaluation in clinical practice.

[13] Rapp AM, Bergman RL, Piacentini J, McGuire JF (2016). Evidence-based assessment of obsessive-compulsive disorder. J Cent Nerv Syst Dis.

[14] Storch EA, Murphy TK, Adkins JW, Lewin AB, Geffken GR, Johns NB (2006). The children's Yale-Brown obsessive-compulsive scale: psychometric properties of child- and parent-report formats. J Anxiety Disord.

[15] Leckman JF, Riddle MA, Hardin MT, Ort SI, Swartz KL, Stevenson J (1989). The Yale Global Tic Severity Scale: initial testing of a clinician-rated scale of tic severity. J Am Acad Child Adolesc Psychiatry.

[16] Storch EA, McGuire JF, Wu MS, Hamblin R, McIngvale E, Cepeda SL (2019). Development and psychometric evaluation of the Children's Yale-Brown Obsessive-Compulsive Scale second edition. J Am Acad Child Adolesc Psychiatry.

[17] McGuire JF, Piacentini J, Storch EA, Murphy TK, Ricketts EJ, Woods DW (2018). A multicenter examination and strategic revisions of the Yale Global Tic Severity Scale. Neurology.

[18] Karolinska Universitetslaboratoriet. https://www.karolinska.se/for-vardgivare/karolinska-universitetslaboratoriet/. Accessed 1 Nov 2020

[19] Brown KD, Farmer C, Freeman GM, Spartz EJ, Farhadian B, Thienemann M (2017). Effect of early and prophylactic nonsteroidal anti-inflammatory drugs on flare duration in pediatric acute-onset neuropsychiatric syndrome: an observational study of patients followed by an academic community-based pediatric acute-onset neuropsychiatric syndrome clinic. J Child Adolesc Psychopharmacol.

