# Supplementary material for: A Two-to-Five Year Follow-Up of a Pediatric Acute-Onset Neuropsychiatric Syndrome Cohort
Source: Child Psychiatry Hum Dev. 2021 Feb 9;53(2):354–64. doi: 10.1007/s10578-021-01135-4 (PMC7870456; doi:10.1007/s10578-021-01135-4)
Supplement: Supplementary file 1 — Electronic supplementary material 1 (DOCX 13 kb) [file 10578_2021_1135_MOESM1_ESM.docx]

**Table S1.** Baseline characteristics in consented participants compared to patients unavailable at follow-up.

|  | | | | | | | | | | | |
| --- | --- | --- | --- | --- | --- | --- | --- | --- | --- | --- | --- |
|  |  |  |  |  |  |  |  |  |  |  |  |
|  | Consented (n=34) | | | | Non-consented (n=12) | | | | Comparison consented vs non-consented | | |
|  |  |  |  |  |  |  |  |  |  |  |  |
|  | n | % | median (range) | mean (SD) | n | % | median (range) | mean (SD) | χ2 | z | p |
| Male | 19 | 56 | . | . | 6 | 50 | . | . | 0.12 | . | 0.73 |
| Age at intake (years) | . | . | 7.7 (3.3-14.2) | 8.2 (2.8) | . | . | 9.7 (3.5-14.4) | 9.2 (3.4) | . | 0.85 | 0.39 |
| Age at disease onset (years) | . | . | 6.6 (3-11.5) | 6.9 (2.1) | . | . | 8.4 (2.8-13.1) | 8.2 (2.9 | . | 1.45 | 0.15 |
| CGAS^a^ at intake | . | . | 53 (24-70) | 51.1 (9.6) | . | . | 52.5 (21-70) | 51.2 (12.6) | . | 0.18 | 0.86 |
| CGI-S^b^ at intake | . | . | 4 (2-6) | 3.8 (0.9) | . | . | 4 (2-6) | 3.8 (1.1) | . | -0.29 | 0.77 |

^a^CGAS: Children’s Global Assessment Scale

^b^CGI-S: Clinical Global Impression – Severity scale
