# Supplementary material for: A Two-to-Five Year Follow-Up of a Pediatric Acute-Onset Neuropsychiatric Syndrome Cohort
Source: Child Psychiatry Hum Dev. 2021 Feb 9;53(2):354–64. doi: 10.1007/s10578-021-01135-4 (PMC7870456; doi:10.1007/s10578-021-01135-4)
Supplement: Supplementary file 2 — Electronic supplementary material 2 (DOCX 16 kb) [file 10578_2021_1135_MOESM2_ESM.docx]

**Table S2.** Patient characteristics in the total cohort and comparing the non-chronic and chronic course groups.

| Patient demographics | | | | | Disease course | | | | | | | | | | |
| --- | --- | --- | --- | --- | --- | --- | --- | --- | --- | --- | --- | --- | --- | --- | --- |
|  | Total (n=34) | | | | Non-chronic course (n=22) | | | | Chronic course (n=12) | | | | Comparison non-chronic vs chronic | | |
|  | n | % | median (range) | mean (SD) | n | % | median (range) | mean (SD) | n | % | median (range) | mean (SD) | χ2 | z | p |
| Male | 19 | 56 | . | . | 13 | 59 | . | . | 6 | 50 | . | . | 0.5 | . | 0.62 |
| Age at follow-up (years) | . | . | 11.5 (6.7-17.1) | 11.7 (2.8) | . | . | 11.9 (6.7-17.1) | 12.3 (2.6) | . | . | 9.7 (7.3-16.4) | 10.5 (2.8) | . | 2.04 | 0.04* |
| Age at disease onset (years) | . | . | 6.6 (3-11.5) | 6.9 (2.1) | . | . | 7.3 (3.6-11.4) | 7.4 (1.8) | . | . | 5.4 (3-11.5) | 5.9 (2.3) | . | 2.36 | 0.02* |
| Time since symptom onset (years) | . | . | 4.8 (3-9.2) | 4.8 (1.5) | . | . | 4.7 (3.1-9.2) | 4.9 (1.6) | . | . | 4.9 (3-8.3) | 4.7 (1.4) | . | 0.47 | 0.64 |
| Follow-up time (years) | . | . | 3.3 (2.3-4.9) | 3.5 (0.7) | . | . | 3.4 (2.3-4.9) | 3.5 (0.7) | . | . | 3.3 (2.4-4.8) | 3.6 (0.9) | . | -0.25 | 0.8 |
| Flares last 12 months | . | . | 1 (0-3) | 1 (1.1) | . | . | 0.5 (0-3) | 0.8 (1) | . | . | 1 (0-3) | 1.3 (1.2) | . | -1.22 | 0.22 |
| Verified infections last 12 months | . | . | 0 (0-4) | 0.6 (0.9) | . | . | 0 (0-2) | 0.5 (0.7) | . | . | 0.5 (0-4) | 0.8 (1.2) | . | -0.86 | 0.39 |
| Unverified infections last 12 months | . | . | 2.5 (0-8) | 2.6 (2) | . | . | 2 (0-6) | 2.4 (1.8) | . | . | 3 (0-8) | 3 (2.3) | . | -0.84 | 0.4 |
| CGAS^a^ at baseline | . | . | 53 (24-70) | 51.1 (9.6) | . | . | 55.5 (24-70) | 53.3 (9.8) | . | . | 48 (30-58) | 47.2 (8.2) | . | 2.08 | 0.04* |
| CGAS^a^ at follow-up | . | . | 61 (28-80) | 60.9 (13.4) | . | . | 68 (32-80) | 66.3 (11.7) | . | . | 54 (28-64) | 51 (10.8) | . | 3.54 | <0.001* |
| CGI-S^b^ at baseline | . | . | 4 (2-6) | 3.8 (0.9) | . | . | 4 (2-6) | 3.6 (0.2) | . | . | 4 (3-5) | 4.1 (0.7) | . | -1.61 | 0.11 |
| CGI-S^b^ at follow-up | . | . | 3 (1-6) | 2.8 (1.4) | . | . | 2 (1-5) | 2.1 (1.1) | . | . | 4 (3-6) | 4 (1) | . | -3.88 | <0.001* |
| CGI-I^c^ | . | . | 1 (1-4) | 1.6 (0.8) | . | . | 1 (1-3) | 1.3 (0.6) | . | . | 2 (1-4) | 2 (1) | . | -2.08 | 0.04* |
| School attendance <80% | 6 | 18 | . | . | 1 | 5 | . | . | 5 | 42 | . | . | 7.36 | . | 0.01* |
| Previous neuropsychiatric diagnose | 2 | 6 | . | . | 1 | 5 | . | . | 1 | 8 | . | . | 0.2 | . | 0.65 |
| Neuropsychiatric diagnose during follow-up | 13 | 38 | . | . | 6 | 27 | . | . | 7 | 58 | . | . | 3.17 | . | 0.08 |
| Comorbid inflammatory/autoimmune disease | 11 | 32 | . | . | 5 | 23 | . | . | 6 | 50 | . | . | 2.64 | . | 0.1 |
| Onset of inflammatory/autoimmune disease during follow-up | 4 | 12 | . | . | 2 | 9 | . | . | 2 | 17 | . | . | 0.43 | . | 0.51 |
| Family history of psychiatric disorder | 27 | 79 | . | . | 18 | 82 | . | . | 9 | 75 | . | . | 0.62 | . | 0.64 |
| Family history of inflammatory disorder/autoimmune disease | 28 | 82 | . | . | 18 | 82 | . | . | 10 | 83 | . | . | 0.01 | . | 0.91 |

^a^CGAS: Children’s Global Assessment Scale

^b^CGI-S: Clinical Global Impression – Severity scale

^c^CGI-I: Clinical Global Impression – Improvement scale
