# Supplementary material for: A Two-to-Five Year Follow-Up of a Pediatric Acute-Onset Neuropsychiatric Syndrome Cohort
Source: Child Psychiatry Hum Dev. 2021 Feb 9;53(2):354–64. doi: 10.1007/s10578-021-01135-4 (PMC7870456; doi:10.1007/s10578-021-01135-4)
Supplement: Supplementary file 3 — Electronic supplementary material 3 (DOCX 14 kb) [file 10578_2021_1135_MOESM3_ESM.docx]

**Table S3.** Symptoms and somatic signs in the total cohort and comparing the non-chronic and chronic course groups.

| Symptoms and somatic signs | | | Disease course | | | | | |
| --- | --- | --- | --- | --- | --- | --- | --- | --- |
|  |  |  |  |  |  |  |  |  |
|  | Total (n=34) | | Non-chronic course (n=22) | | Chronic course (n=12) | | Comparison non-chronic vs chronic | |
|  |  |  |  |  |  |  |  |  |
|  | n | % | n | % | n | % | χ2 | p |
| Obsessive-compulsive symptoms | 21 | 62 | 11 | 50 | 10 | 83 | 3.65 | 0,06 |
| CY-BOCS^a^ >15 | 5 | 15 | 2 | 9 | 3 | 25 | 1.57 | 0.21 |
| Tics | 17 | 50 | 10 | 45 | 7 | 58 | 0.52 | 0.47 |
| YGTSS^b^ >30 | 2 | 6 | 0 | 0 | 2 | 17 | 3.9 | 0.05* |
| Hyperactivity/impulsivity | 12 | 35 | 3 | 14 | 9 | 75 | 12.8 | <0.001* |
| Anxiety | 12 | 35 | 4 | 18 | 8 | 67 | 7.99 | 0.01* |
| Behavioral difficulties | 11 | 32 | 4 | 18 | 7 | 58 | 5.72 | 0.02* |
| Depressive symptoms | 10 | 29 | 3 | 14 | 7 | 58 | 7.47 | 0.01* |
| Sleeping disorder | 10 | 29 | 2 | 9 | 8 | 67 | 12.4 | <0.001* |
| Tiredness/fatigue | 10 | 29 | 5 | 23 | 5 | 42 | 1.34 | 0.25 |
| Pain | 8 | 24 | 3 | 14 | 5 | 42 | 3.39 | 0.07 |
| Cognitive difficulties | 6 | 18 | 3 | 14 | 3 | 25 | 0.69 | 0.41 |
| Eating disorder | 5 | 15 | 1 | 5 | 4 | 33 | 5.13 | 0.02* |
| Urinary problems | 2 | 6 | 0 | 0 | 2 | 17 | 3.9 | 0.05* |
| Perceived changes in personality | 1 | 3 | 0 | 0 | 1 | 8 | 1.89 | 0.17 |
| Abnormalities in somatic assessment | 33 | 36 | 6 | 29 | 6 | 50 | 1.52 | 0.22 |

^a^CY-BOCS: Children’s Yale-Brown Obsessive Compulsive Scale

^b^YGTSS: Yale Global Tic Severity Scale
