# Supplementary material for: A Two-to-Five Year Follow-Up of a Pediatric Acute-Onset Neuropsychiatric Syndrome Cohort
Source: Child Psychiatry Hum Dev. 2021 Feb 9;53(2):354–64. doi: 10.1007/s10578-021-01135-4 (PMC7870456; doi:10.1007/s10578-021-01135-4)
Supplement: Supplementary file 4 — Electronic supplementary material 4 (DOCX 14 kb) [file 10578_2021_1135_MOESM4_ESM.docx]

**Table S4.** Pharmacological and psychological interventions in the total cohort and comparing the non-chronic and chronic course groups.

| Interventions last 12 months | | | Disease course | | | | | |
| --- | --- | --- | --- | --- | --- | --- | --- | --- |
|  | Total (n=34) | | Non-chronic course (n=22) | | Chronic course (n=12) | | Comparison non-chronic vs chronic | |
|  |  |  |  |  |  |  |  |  |
|  | n | % | n | % | n | % | χ2 | p |
| Medication, in total | 29 | 85 | 17 | 77 | 12 | 100 | 3.3 | 0.07 |
| Melatonine | 16 | 47 | 7 | 24 | 9 | 75 | 5.81 | 0.02* |
| NSAIDs^a^ | 15 | 44 | 6 | 27 | 9 | 75 | 7.17 | 0.01* |
| Antibiotics | 14 | 41 | 6 | 27 | 8 | 67 | 4.97 | 0.03* |
| Guanfacine | 8 | 24 | 1 | 5 | 7 | 58 | 12.49 | <0.001* |
| SSRI^b^ | 7 | 21 | 5 | 23 | 2 | 17 | 0.17 | 0.68 |
| Cortisone | 4 | 12 | 1 | 5 | 3 | 25 | 3.13 | 0.08 |
| IVIG^c^ | 4 | 12 | 0 | 0 | 4 | 33 | 8.31 | <0.001* |
| Stimulants | 3 | 9 | 3 | 14 | 0 | 0 | 1.79 | 0.18 |
| Neuroleptics | 1 | 3 | 0 | 0 | 1 | 9 | 1.89 | 0.17 |
| Psychological treatments/interventions, in total | 20 | 59 | 10 | 45 | 10 | 83 | 4.6 | 0.03* |
| CBT^d^ | 13 | 38 | 8 | 36 | 5 | 42 | 0.09 | 0.76 |
| Other interventions (parental strategies, groups, counseling) | 15 | 44 | 6 | 27 | 9 | 75 | 7.17 | 0.01* |

^a^NSAIDs: Non-Steroidal Anti-inflammatory Drugs

^b^SSRI: Selective Serotonine Re-uptake Inhibitor

^c^IVIG: Intra-Venous Immunoglobulines

^d^CBT: Cognitive Behavioral Therapy
