# Supplementary material for: A Two-to-Five Year Follow-Up of a Pediatric Acute-Onset Neuropsychiatric Syndrome Cohort
Source: Child Psychiatry Hum Dev. 2021 Feb 9;53(2):354–64. doi: 10.1007/s10578-021-01135-4 (PMC7870456; doi:10.1007/s10578-021-01135-4)
Supplement: Supplementary file 5 — Electronic supplementary material 5 (DOCX 16 kb) [file 10578_2021_1135_MOESM5_ESM.docx]

**Table S5.** Laboratory test results in the total cohort and across disease course and symptom burden groups.

| Laboratory tests | | | Disease course | | | | | | PANS symptom burden | | | | | |
| --- | --- | --- | --- | --- | --- | --- | --- | --- | --- | --- | --- | --- | --- | --- |
|  |  |  |  |  |  |  |  |  |  |  |  |  |  |  |
|  |  |  |  |  |  |  |  |  |  |  |  |  |  |  |
|  | Total (n=27) | | Non-chronic course (n=22) | | Chronic course (n=12) | | Comparison non-chronic vs chronic | | Asymptomatic | | Current PANS-symptoms | | Comparison asymptomatic vs current symptoms | |
|  |  |  |  |  |  |  |  |  |  |  |  |  |  |  |
|  | n | % | n | % | n | % | χ2 | p | n | % | n | % | χ2 | p |
| Protein fractions abnormalities (incl CRP) | 20 | 74 | 13 | 72 | 7 | 78 | 0.1 | 0.76 | 7 | 64 | 13 | 81 | 1.05 | 0.31 |
| CBC^a^ abnormalities | 18 (n total=26) | 69 | 14 | 78 | 4 | 50 | 2 | 0.16 | 7 | 64 | 11 | 73 | 0.28 | 0.6 |
| IgG^b^ sub-class deficiencies | 11 | 41 | 6 | 33 | 5 | 56 | 1.23 | 0.27 | 6 | 55 | 5 | 31 | 1.47 | 0.23 |
| Elevated IL-1-β^c^ | 9 (n total=25) | 36 | 3 | 18 | 6 | 75 | 7.77 | 0.01* | 1 | 10 | 8 | 53 | 4.89 | 0.03* |
| Complement abnormalities | 6 | 22 | 5 | 28 | 1 | 11 | 0.96 | 0.33 | 4 | 36 | 2 | 13 | 2.15 | 0.14 |
| Low vitamin D | 6 | 22 | 4 | 22 | 2 | 22 | 0 | 1 | 3 | 27 | 3 | 19 | 0.27 | 0.6 |
| Low IgA^d^ | 6 | 22 | 3 | 17 | 3 | 33 | 0.96 | 0.33 | 1 | 9 | 5 | 31 | 1.85 | 0.17 |
| Elevated IL-10^e^ | 5 (n total=25) | 20 | 2 | 12 | 3 | 38 | 5.72 | 0.46 | 1 | 10 | 4 | 27 | 1.04 | 0.31 |
| Elevated TNF-α^f^ | 5 (n total=26) | 19 | 0 | 0 | 5 | 56 | 11.69 | <0.001* | 0 | 0 | 5 | 31 | 3.87 | 0.05* |
| Elevated anti-TPO^g^ | 5 | 19 | 3 | 17 | 2 | 22 | 0.12 | 0.73 | 3 | 27 | 2 | 13 | 0.94 | 0.33 |
| TSH^h^ abnormalities | 4 | 15 | 3 | 17 | 1 | 11 | 0.15 | 0.70 | 2 | 18 | 2 | 13 | 0.17 | 0.68 |
| Positive throat culture | 3 (n total=25) | 12 | 2 | 12 | 1 | 13 | 0 | 0.96 | 0 | 0 | 3 | 21 | 2.68 | 0.1 |
| Low T4^i^ | 3 | 11 | 2 | 11 | 1 | 11 | 0 | 1 | 0 | 0 | 3 | 19 | 2.32 | 0.13 |
| Elevated IgM^j^ | 3 | 11 | 0 | 0 | 3 | 33 | 6.75 | 0.01* | 0 | 0 | 3 | 19 | 2.32 | 0.13 |
| Low ferritin | 3 | 11 | 1 | 6 | 2 | 22 | 1.69 | 0.19 | 0 | 0 | 3 | 19 | 2.32 | 0.13 |
| Positive ANA^k^ | 2 | 7 | 1 | 6 | 1 | 11 | 0.27 | 0.6 | 0 | 0 | 2 | 13 | 1.49 | 0.22 |
| Elevated ALAT^l^ | 2 | 7 | 1 | 6 | 1 | 11 | 0.27 | 0.6 | 1 | 9 | 1 | 6 | 0.08 | 0.78 |
| Elevated ESR^m^ | 1 (n total=26) | 4 | 0 | 0 | 1 | 13 | 2.34 | 0.13 | 0 | 0 | 1 | 7 | 0.76 | 0.76 |
| Elevated CRP^n^ | 1 | 4 | 1 | 6 | 0 | 0 | 0.52 | 0.47 | 0 | 0 | 1 | 6 | 0.71 | 0.4 |
| Elevated SAA^o^ | 1 | 4 | 1 | 6 | 0 | 0 | 0.52 | 0.47 | 0 | 0 | 1 | 6 | 0.71 | 0.4 |
| Low IgG^b^ | 1 | 4 | 0 | 0 | 1 | 11 | 2.08 | 0.15 | 0 | 0 | 1 | 6 | 0.71 | 0.4 |
| Cystatin C abnormalities | 0 | 0 | 0 | 0 | 0 | 0 | . | . | 0 | 0 | 0 | 0 | . | . |
| Elevated transglutaminase antibodies | 0 | 0 | 0 | 0 | 0 | 0 | . | . | 0 | 0 | 0 | 0 | . | . |
| Elevated IL-6^p^ | 0 (n total=26) | 0 | 0 | 0 | 0 | 0 | . | . | 0 | 0 | 0 | 0 | . | . |
| Elevated IL-8^q^ | 0 (n total=26) | 0 | 0 | 0 | 0 | 0 | . | . | 0 | 0 | 0 | 0 | . | . |

^a^CBC: Complete Blood Count

^b^IgG: Immunoglobulin G

^c^IL-1-β: Interleukin 1 β

^d^IgA: Immunoglobulin A

^e^IL-10: Interleukin 10

^f^TNF-α: Tumor Necrosis Factor α

^g^Anti-TPO: Thyroid Peroxidase Antibodies

^h^TSH: Thyroid Stimulating Hormone

^i^T4: Thyroxine

^j^IgM: Immunoglobulin M

^k^ANA: Antinuclear Antibodies

^l^ALAT: Alanine Aminotransferase

^m^ESR: Erythrocyte Sedimentation Rate

^n^CRP: C-reactive Protein

^o^SAA: Serum Amyloid A

^p^IL-6: Interleukin 6

^q^IL-8: Interleukin 8
